# Examining the Complex Mismatch Negativity in Early Phase Psychosis Using the Dual Rule Paradigm

**DOI:** 10.1177/15500594241273287

**Published:** 2024-08-16

**Authors:** Jenna N. Bissonnette, T-Jay Anderson, Candice E. Crocker, Philip G. Tibbo, Dean F. Salisbury, Derek J. Fisher

**Affiliations:** 1 Department of Psychiatry, 3688 Dalhousie University, Halifax, Nova Scotia, Canada; 2 Department of Psychology & Neuroscience, 3688 Dalhousie University, Halifax, Nova Scotia, Canada; 3 Department of Psychiatry, University of Pittsburgh, Pittsburgh, Pennsylvania, USA; 4 Department of Psychology, 3684 Mount Saint Vincent University, Halifax, Nova Scotia, Canada

**Keywords:** complex mismatch negativity, early phase psychosis, auditory hallucinations, electroencephalography, biomarker

## Abstract

Using electroencephalography (EEG) to examine the simple mismatch negativity (MMN), a marker of auditory cortex function, has been of great interest in the exploration of biomarkers for psychotic illness. Despite many studies reporting MMN deficits in chronic schizophrenia, there are inconsistent reports of MMN reductions in the early phases of psychotic illness, suggesting the MMN elicited by traditional paradigms may not be a sensitive enough measure of vulnerability to be used as a biomarker. Recently, a more computationally complex measure of auditory cortex function (the complex mismatch negativity; cMMN) has been hypothesized to provide a more sensitive marker of illness vulnerability. The current study employed a novel dual rule paradigm, in which two pattern rules are established and violated, to examine the cMMN in 14 individuals with early phase psychosis (EPP, < 5 years illness) and 15 healthy controls (HC). Relationships between cMMN waveforms, symptom severity, and measures of functioning were explored. We found reductions of cMMN amplitudes at the site of maximal amplitude in EPP (*p *= .017) with large effect sizes (*Hedges’ g *= 0.96). This study is an early step in the exploration of the cMMN as a biomarker for psychosis. Our results provide evidence that the dual rule cMMN paradigm shows promise as a method for cMMN elicitation that captures more subtle neurofunctional changes in the early stages of illness.

Early intervention and a shorter duration of untreated psychosis during the early phase of psychosis (the first five years following symptom onset; EPP) can greatly improve long term outcomes including antipsychotic treatment response as well as functional outcomes.^1,2^ Accordingly, identifying individuals who are at-risk as early as possible and providing interventions are critical.^3^ Before the presence of symptoms that exceed a clinical threshold, prodromal symptoms can classify someone as being clinically at-risk (CHR). However, only approximately 21%–36% of CHR individuals will go on to develop psychosis.^4,5^ This underscores the need for a more sensitive tool to identify at-risk individuals for psychosis development than just symptoms alone, as 70%–80% of CHR individuals are not prodromal for schizophrenia.

Electroencephalography (EEG) derived event-related potentials (ERPs) are a direct and objective measure of neuronal functioning.^6^ The mismatch negativity (MMN) is an ERP with a negative polarity that is generated in the frontotemporal regions of the cortex and can be recorded at frontal electrode sites (ie, site of maximal amplitude at Fz) approximately 100–250 milliseconds following a detectable change in the auditory environment,^7^ and can be conceptualized as a general indicator of auditory cortex function.^8^ The critical involvement and dependence of MMN generation on N-methyl-D-aspartate (NMDA) receptors link this response to the glutamatergic system.^9,10^ Glutamate alterations have been reported across the phases of psychotic illness including the chronic phase,^11^ early phase,^12,13^ and in some CHR samples^14^ which highlights the potential for MMN alterations in these populations.

To date, MMN reductions to tones that deviate in duration have been robustly reported in chronic schizophrenia samples.^15^ This reduction is correlated with increased illness duration,^16^ psychosis symptomology,^17,18^ and impaired daily functioning.^19^ This duration MMN reduction has been reported in some CHR samples, with those converting to psychosis showing more established reductions, and with other studies reporting no differences in the duration MMN response of CHR samples or between converters and non-converters.^20–23^ However, in early presentations of the illness, the magnitude of a duration MMN reduction is less robust,^15^ and reports of no duration MMN reductions in EPP samples exist.^24,25^ If a duration MMN reduction was truly a sensitive marker of illness vulnerability, robust reductions would be present at the early phase of the illness. Therefore, the exploration of other ways to probe these early sensory processes that are better suited to capture subtle changes in cortical function have become of interest.

One of these approaches has been to explore different paradigms to elicit the MMN that require more complex computational processes (eg identifying deviance in multiple patterns) have been explored. The MMN in response to these paradigms is referred to as a complex MMN (cMMN) and is hypothesized to rely on higher-order cognitive processing (relative to the traditional MMN response) that employs the frontal cortex as well as the primary auditory cortex.^26–28^ Due to this computational complexity, it is hypothesized that the cMMN will deteriorate earlier in illness progression compared to the traditional MMN response, and will therefore provide a higher sensitivity in detecting deficits early on in illness progression.

To date, paradigms to elicit the cMMN have involved either deviant tones that vary from a standard in two physical attributes concurrently (ie, complex tone paradigms) or violate an established pattern (ie, complex pattern paradigms). While complex tone paradigms have shown greater sensitivity to reductions at earlier phases of illness progression,^29,30^ a recent meta-analysis synthesized the results from complex tone and complex pattern paradigms together and found similar effect sizes to those reported from traditional MMN paradigms.^26^ However, when examining only complex pattern paradigms, studies consistently report cMMN reductions in chronic and first episode psychosis samples.^27,31–33^ This suggests the optimal method of eliciting the cMMN may lie within complex pattern paradigms, and that cMMN reductions elicited by these paradigms are more robustly reduced across the illness course compared to the traditional MMN response.

## The Current Study

**Figure 1.**
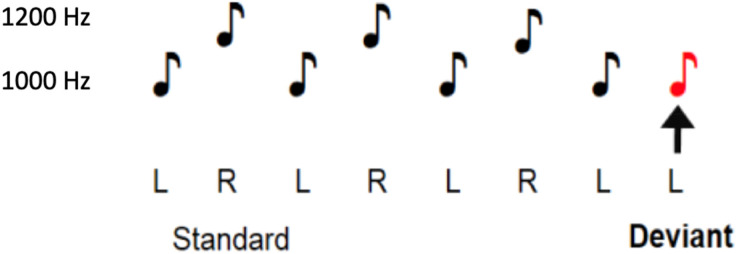
Visual representation of the dual rule complex MMN paradigm*. Note.* This figure represents the auditory tones played during the dual rule cMMN paradigm. Music notes above a “L” indicate they were played to the left ear, while music notes above a “R” indicate they were played to the right ear. Music notes next to the “1200 Hz” were high-frequency tones while music notes next to the “1000 Hz” low frequency tones. The red music note represents the deviant tone.

The aim of the current study was to examine a novel cMMN paradigm in EPP individuals compared to healthy controls. This new paradigm, created by Dr Dean Salisbury at the University of Pittsburgh and hereby referred to as the dual rule complex paradigm, breaks two pattern rules simultaneously by establishing a pattern of low-frequency tones played to the left ear, and high-frequency tones played to the right. The deviant tone that elicits the cMMN is a repeated tone in the pattern, thus breaking both the right/left alternating pattern and the high/low-frequency pattern while generating no confounding release from repetition suppression, as the deviant is physically identical to the preceding sound (see Figure 1). Conceptually, adding an additional pattern rule to a paradigm requires an increased computational demand on the cortex to hold two pattern rules in sensory memory, and then detect the deviation from those two rules. This increased demand on the cortex was therefore hypothesized to be advantageous in detecting more subtle deficits that occur early on in illness progression. Also included in this special issue is a description of this novel cMMN paradigm in healthy individuals by Salisbury and colleagues.

## Methods

### Participants

**Figure 2.**
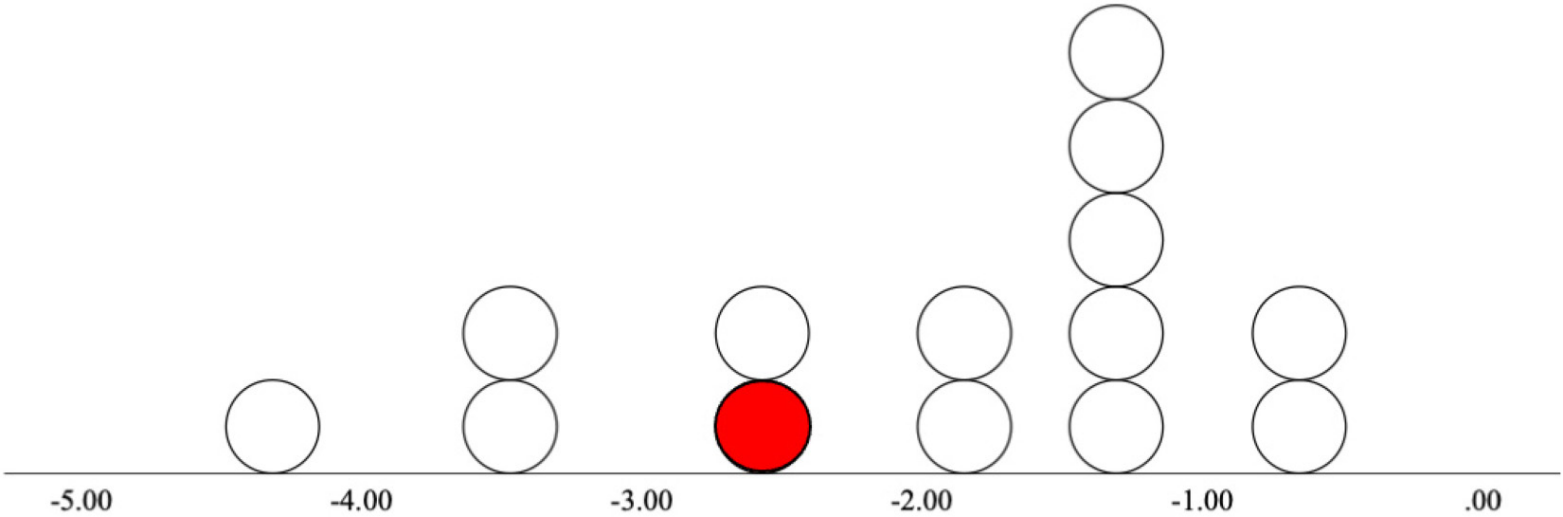
Distribution of cMMN waveform amplitudes at electrode site Fz in EPP group. Note. This figure demonstrates the distribution of cMMN waveform amplitudes (µV) at Fz for each participant in the early phase psychosis (EPP) group. The one individual diagnosed with substance-induced psychosis is represented in red.

A sample of 14 individuals (9 males, 5 females) between the ages of 20–26 (*M_age _*= 22.9, *SD_age _*= 2.2) who were within the first five years of admission to the Nova Scotia Early Psychosis Program (NSEPP) were recruited. The specific diagnoses of the EPP group were as follows: seven individuals with schizophrenia, three individuals with unspecified schizophrenia spectrum disorder, two individuals with schizoaffective disorder, one individual with substance-induced psychosis (see Figure 2), and one individual with schizophreniform disorder. 15 Healthy controls (HC) were recruited from the general population through electronic and paper advertisements and through word of mouth (4 males, 11 females; between the ages of 18–26 [*M_age _*= 23.1, *SD_age _*= 2.5]). HCs had no past or current mental illness diagnosis and had no first-degree relatives with psychosis, as alterations of MMN have been observed in unaffected immediate family members of those with schizophrenia.^34^ Participants were excluded if they met any of the following criteria: self-reported co-morbid DSM-V disorder; a history of significant head injury resulting in loss of consciousness within the past year; diagnosis of epilepsy or any other neurologic disorder; electro-convulsive therapy (ECT) treatment within the previous year; significant cardiac illness; chronic medical illness requiring regular medication; extrapyramidal symptoms (EPS) resulting in movement disorders; abnormal audiometric assessment; or left-handedness.

### Questionnaires

#### Global Assessment of Functioning Scale

The Global Assessment of Functioning Scale (GAF) rates the social and occupational functioning of adults on a scale from 0–100 (where higher scores indicate higher functioning) and has shown to have adequate reliability as well as validity in clinical populations.^35^

#### Scale of Prodromal Symptoms

The Scale of Prodromal Symptoms (SOPS) is a 19-item scale designed to assess prodromal symptoms of psychosis in high-risk populations.^36^ The current study utilized two SOPS subscales of positive and negative symptoms. SOPS scores are highly correlated with the Positive and Negative Syndrome Scale (PANSS) scores and the SOPS has previously been employed in an EPP sample to assess the degree of psychosis symptoms.^37^

#### Psychotic Symptoms Rating Scales

The AH-subscale of the Psychotic Symptoms Rating Scales (PSYRATS)^38^ was used to assess auditory hallucinations in our EPP group. The PSYRATS has also been validated in a sample of individuals in the early phase of psychosis, and complements additional measures of psychosis symptomology by probing a detailed profile of auditory hallucination dimensions.^39^ The PSYRATS can be divided into four subscales including distress, frequency, attribution, and loudness of auditory hallucinations.^40^

### Procedure

EPP patients were approached by their clinician and provided verbal consent to be contacted for screening. A brief screening questionnaire was completed over the phone to determine the eligibility of the participant. At the same time as the screening, demographic variables of age, sex, and level of education were obtained. Additionally, due to the reported associations between substance use and MMN alterations,^41,42^ self-reported weekly average alcohol and cannabis consumption were obtained for group comparisons. If the volunteer met all inclusion criteria and was eligible to participate, they scheduled one session in the lab (at Halifax, Nova Scotia, Canada). All sessions were completed between the hours of 12:00 pm to 3:00 pm to account for circadian fluctuations in alertness and EEG patterns throughout the day.^43^ Participants were required to abstain from alcohol, cannabis, and illicit substances from midnight the night prior to the session. Participants in the EPP group were instructed to take their medications (including antipsychotic and adjunct medications) as usual. Upon arrival to the session, verbal confirmation of abstinence was obtained from each participant.

Once arrived at the lab, following informed consent procedures, the participant completed all relevant questionnaires for their experimental group. Following the completion of the questionnaires, EEG electrodes were applied. Participants were instructed to watch an emotionally neutral silent film while a battery of auditory MMN paradigms was presented that included the dual rule cMMN paradigm.

### Dual Rule Paradigm Parameters

The dual rule cMMN paradigm consists of tones that alternate between presentation to the left and right ear, as well as between high and low frequencies. The standard trial pattern consists of the low frequency tone played to the left ear, then the high frequency tone played to the right ear. Deviant trials consist of at least two presentations of the low-left, high-right pattern, followed by the low frequency tone played to the left ear twice (ie a repetition of the first tone in the standard pattern, or a tone that fails to switch both frequency and location, thus breaking the two established patterns of left/right and high/low frequency). High-frequency tones were composed of 1200 Hz pure tones delivered to the right ear. Low-frequency tones were composed of 1000 Hz pure tones delivered to the left ear. Both tones were 75 ms in length with 5 ms rise and fall. The interstimulus interval was 330 ms. In total 1200 tones were presented. Standard trials were presented six times before a deviant trial resulting in deviant tones contributing to 11% of the presented stimuli (132 deviant tone presentations). The stimuli were presented through headphones at an intensity of 75 dB SPL in one 8-min interval.

### EEG Recording Parameters and cMMN Computation

EEG recording was digitally sampled at 500 Hz from an active electrode cap with Ag+/Ag + -Cl- electrodes at 64 scalp sites. Scalp sites were chosen according to the 10–10 system of electrode placement. Relevant data was collected from electrode placements at two midline sites (frontal [Fz], central [Cz]), two right hemisphere (frontal [F4], central [C4]) electrode sites, and two left hemisphere (frontal [F3], central [C3]) electrode sites. Electrodes were also placed bilaterally on both mastoids. During recording, electrode site Fz served as reference and Fpz served as ground. The data was then re-referenced to bilateral mastoid sites. Recordings of vertical electrooculogram activity was taken from Fp1/Fp2. All electrode impedances were kept under 10 kΩ at the time of recording. Electrical activity was recorded using an ActiCHamp (Brain Products, Gilching, NE) with a bandpass filter of DC-250 Hz, and stored on a hard disk for later off-line analysis.

Offline EEG data processing included first applying IIR filters from 0.1–20 Hz to all electrode sites except Fp1 and Fp2, which were filtered from 0.1–3 Hz to enhance electrooculogram activity at these sites to be used for later ocular correction. Then, segmentation relative to each deviant tone was completed including 100 ms before and 700 ms after each tone. An ocular correction was then completed using a well-established procedure^44^ followed by a baseline correction starting 100 ms pre-stimulus. Artifact rejection was done for any epoch exceeding 50 µV. Finally, separate averages were taken for standard and deviant ERPs within each participant.

The cMMN difference waveforms were derived by digital point-by-point subtraction of the values from the standard stimulus presentations (ie, any stimulus that was not a deviant tone) from those elicited by the presentation of the deviant stimuli. cMMN peaks were assessed by an automatic detection of peak negative amplitudes (relative to average pre-stimulus baseline activity) within a 100–250 milliseconds post-stimulus analysis window. The automatized peak detection was then confirmed based on visual inspection of the waveform. The output was the average electrical activity ± 8 ms of the peak amplitude.

### Statistical Analysis

All statistical analyses were carried out using the Statistical Package for the Social Sciences (SPSS version 27; IBM Corp., Armonk NY). Demographic variables of age, level of education, and weekly self-reported alcohol and cannabis use measures were compared between groups using independent samples t-tests (equal variances assumed). cMMN amplitudes were subjected to separate general linear model (GLM) mixed measures analyses of variance (ANOVA). Each ANOVA had the between-subjects factor of group (2 levels: HC, EPP) and two within-subject factors of region (2 levels: frontal, central) and laterality (3 levels: right, midline, left). This allowed us to examine cMMN amplitude at regions, lateralities, and single electrode sites (F3, Fz, F4, C3, Cz, C4) separately. Follow-up analyses of significant (p < .05; Bonferroni-corrected) main or interaction effects found in the ANOVAs were carried out with pairwise comparisons using separate (vs pooled) error estimates.

## Results

### Demographic Variables

HC and EPP groups did not vary significantly in age, level of education, or weekly reported alcohol or cannabis consumption (see Table 1). The HC group had a higher percentage of females than males (73% female) compared to the EPP group which had a higher percentage of males than females (36% female). The average SOPS scores for each subscale ranged from 0.6 to 2.2 (out of a possible 6). This indicates a mild symptom severity on average in our EPP sample that aligns with expectations for an outpatient group (see Table 2).

**Table 1.** Group Comparison of Demographic Variables Used in the cMMN Analysis.

	EPP (*n *= 14)	HC (*n *= 15)	*t*(df)	*p*-value
Age (years)	22.9 (±2.2)	23.1 (±2.5)	t(27) = 0.236	.816
Sex (M/F)	9/5	4/11		
Level of education (years)	12.9 (±2.8)	14.6 (±2.1)	t(27) = 1.811	.081
Weekly alcohol consumption (drinks per week)	3.3 (±5.5)	3.5 (±3.0)	t(27) = 0.078	.938
Weekly cannabis consumption (times consumed per week)	2.8 (±3.1)	1.2 (±2.3)	t(27) = -1.554	.132

*Note.* This table displays the mean (± standard deviation) values for demographic variables collected in both the healthy control (HC) and early phase psychosis (EPP) groups for the cMMN analysis.

** Indicates a statistically significant difference between groups on an independent samples t-test at the *p *< .05 level.

**Table 2.** Clinical Variables Used in the cMMN Analysis.

	M (±SD)
**Medication Status** (medicated/non-medicated)	(13/1)
**GAF**	67.5 (±22.0)
**PSYRATS**	
Total	13.8 (±12.3)
Distress	7.0 (±6.9)
Frequency	2.9 (±2.8)
Attribution	2.6 (±2.2)
Loudness	1.6 (±1.6)
**SOPS**	
Unusual thought content/delusional ideas (P1)	2.1 (±1.9)
Suspiciousness/persecutory ideas (P2)	2.1 (±1.5)
Grandiose ideas (P3)	0.6 (±1.3)
Perceptual abnormalities/hallucinations (P4)	2.2 (±1.8)
Disorganized communication (P5)	1.6 (±1.7)
Social Anhedonia (N1)	1.9 (±1.9)
Avolition (N2)	1.6 (±1.6)
Expression of emotion (N3)	1.0 (±1.2)
Experience of emotions and self (N4)	1.4 (±1.6)
Ideational richness (N5)	0.9 (±0.8)
Occupational functioning (N6)	2.1 (±2.4)

*Note.* Average (±standard deviation) scores on the Global Assessment of Functioning Scale (GAF), Psychotic Symptoms Rating Scale (PSYRATS), and Scale of Prodromal Symptoms (SOPS) in the early phase psychosis sample used in the cMMN analysis (*n *= 14).

### cMMN Amplitudes

**Figure 3.**
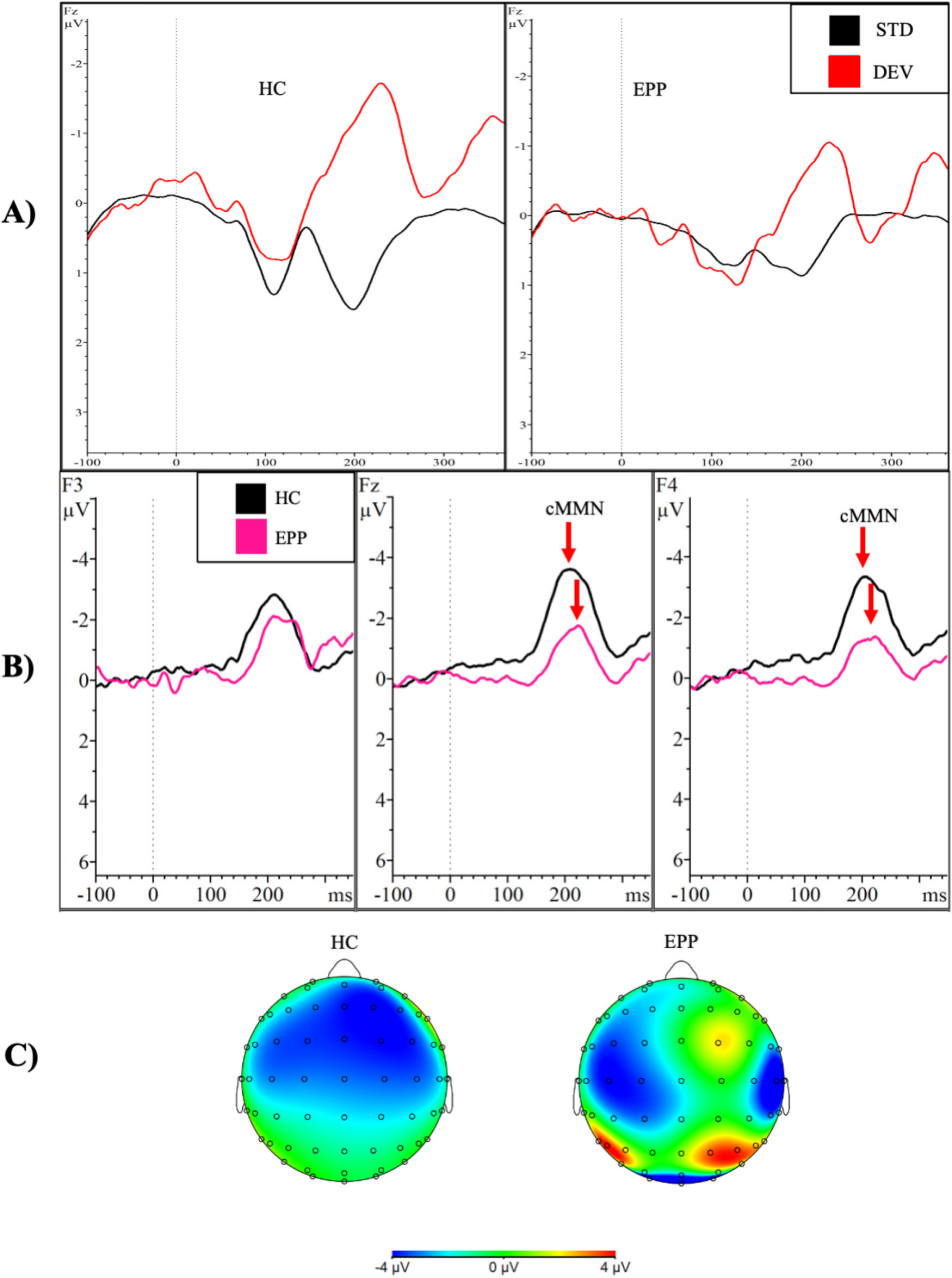
Group comparison of cMMN response from dual rule paradigm*. Note.* This figure demonstrates (A) event-related potential waveforms in response to the standard (black) and deviant (red) tones separated by group; (B) the average cMMN amplitudes (standard waveform subtracted from deviant waveform) in the frontal region in response to the deviant tone in the dual rule cMMN paradigm. The black line represents the healthy control group (HC), and the pink line represents the early phase psychosis group (EPP). Statistically significant different amplitudes (*p *< .05) at F4 and Fz are marked with the red arrow; (C) scalp topography maps of the cMMN to the deviant tone in both groups.

There was a significant main effect of site where cMMN amplitudes were higher over midline sites (*M *= -3.15µV, *SD *= 3.12) compared to left hemisphere sites (*M *= -2.46µV, *SD *= 2.71) across both EPP and HC groups (F,[2,26] = 12.843, *p *= .003, *d *= 0.24). A planned pairwise comparison showed a significant group-by-region interaction where the HC group (*M *= -3.43µV, *SD *= 2.68) had higher cMMN amplitudes compared to the EPP group (*M *= -1.85µV, *SD *= 1.36) at the frontal region (*p *= .033, *d *= 0.74) but this was not significant at the central region (*p *= .178). Further planned pairwise comparisons revealed significant group-by-region-by-site interactions where there were higher cMMN amplitudes in the HC group (*M_F4 _*= -3.45µV, *SD_F4 _*= 3.10; *M_Fz _*= -3.91µV, *SD_Fz _*= 2.57) compared to the EPP group (*M_F4 _*= -1.63µV, *SD_F4 _*= 1.21; *M_Fz _*= -2.01µV, *SD_Fz _*= 1.12) at F4 (*p *= .050, *d *= 0.77) and Fz (*p *= .017, *d *= 0.96) electrode sites (see Figure 3; Table 3).

**Table 3.** Group Comparison of Mean Amplitudes and Latencies from the Complex Dual Rule MMN Paradigm.

Electrode Site	Group	Mean (±SD) (µV)	*df*	Mean Square	*F*	*p*-value
F3	HC	−2.92 (±2.14)	1, 27	7.465	1.951	.174
	EPP	−1.90 (±1.74)				
Fz	HC	−3.91 (±2.6)	1, 27	26.251	6.501	.017*
	EPP	−2.01 (±1.1)				
F4	HC	−3.45 (±3.1)	1, 27	23.991	4.222	.050*
	EPP	−1.63 (±1.2)				
C3	HC	−3.11 (±2.9)	1, 27	10.282	0.972	.333
	EPP	−1.92 (±3.6)				
Cz	HC	−4.02 (±3.2)	1, 27	13.213	0.923	.345
	EPP	−2.67 (±4.3)				
C4	HC	−2.87 (±3.5)	1, 27	20.681	2.852	.103
	EPP	−1.18 (±1.5)				
		Mean (ms)	*df*	95% *CI*	*t*	*p*-value
Latency (Fz)	HC	207.73	27	−23.11, 6.29	−1.174	.251
	EPP	216.14				

*Note.* All values for cMMN amplitude are denoted in microvolts (µV). All values for latencies are denoted in milliseconds (ms). * *p* < .05.

### Latency

There were no significant differences in cMMN latencies between HC and EPP groups.

## Discussion

The current study was the first to examine the cMMN response elicited by a novel dual rule cMMN paradigm in a sample of individuals with EPP. Compared to HC, we found an overall reduction of cMMN amplitudes in the frontal region, as well as statistically significant reductions in amplitude with a large effect size at the site of maximal amplitude (Fz; *d *= 0.96) and at F4 (*d *= 0.77) in EPP. This lateralization of effect is not especially unexpected given the nature of tones used in the paradigm. While the processing of phonetic tones is typically lateralized to the left hemisphere, the processing of pure tones has been shown to lateralize to the right hemisphere.^45^ However, it is also possible that the lateralized effect may be in part due to the presentation of deviant tones consistently at the left ear. Future studies should consider counterbalancing the presentation of the deviant tones within the paradigm to further explore this effect.

Although this was the first study to employ the dual rule cMMN paradigm, these reported reductions are similar to previous studies that have found reduced cMMN amplitudes in EPP using different paradigms to elicit the cMMN response.^30–32^ Notably, one previous study that used a pattern cMMN paradigm that alternated between high and low pitch tones without alternating laterality did not find amplitude reductions in a chronic schizophrenia sample,^46^ suggesting the addition of alterating laterality of tone presentation (and thus the violation of two pattern rules simultaneously) may indeed be resulting in a more complex computational load which is providing a more sensitive measure of MMN deficits in early phase psychosis. Moreover, the effect size viewed in the current study does appear to be superior to those reported in meta-analyses of cMMNs elicited by complex tone and pattern paradigms across the illness phase (*d *= 0.59)^26^ as well as traditional MMNs elicited by duration (*d *= 0.47) and pitch (*d *= 0.04) deviants in first episode samples.^47^ However, this singular effect size does not directly compare to those derived by meta-analyses. More studies exploring this paradigm across the illness phase are required to confirm this hypothesis.

The underlying neural correlates of this deficit in the cMMN response likely lie within the well-established neurological abnormalities of schizophrenia. Specifically, widespread cortical grey matter loss and reduced cortical thickness in the temporal region and decreased dendritic spine density in the dorsolateral prefrontal cortex.^48^ Due to the abnormalities in these regions, individuals experiencing psychosis may lack the cortical infrastructure necessary to complete the complex pattern predictions required to generate a cMMN response. Moreover, Rasser et al (2011) found reduced grey matter in the temporal and frontal cerebral regions was related to reduced MMN amplitude in response to frequency and duration deviants in patients with chronic schizophrenia,^19^ further supporting the idea that these illness-related changes in cortical structure are underlying the inability to produce a strong cMMN response.

Additionally, widespread dysfunction of the glutamatergic system may also underlie these cMMN deficits. The current assumption is that similar to the traditional MMN response, the cMMN is dependent on NMDA receptors and the glutamatergic system. Due to the potential alterations of glutamate levels in EPP (reviewed in Bissonnette et al, 2022), the inability to generate a strong cMMN response may be related to an alteration of glutamatergic activity in the auditory cortex. Future studies examining the link between glutamate and the cMMN response in EPP are needed to confirm this assumption.

### Limitations and Future Directions

The current sample size was modest and these findings must be replicated with a larger sample. Additionally, we relied on self-report retrospective measures of cannabis and alcohol use that were likely sensitive to bias. A more in-depth measure of substance use (such as the timeline follow-back method) would have provided a more reliable measure of substance use in this population. Moving forward, future studies should employ the dual rule cMMN paradigm in a sample of individuals within the first episode of psychosis. Additionally, future studies should consider using fMRI and H-MRS measures alongside the dual rule cMMN paradigm to determine the exact location of generation and links with glutamate levels.

### Conclusion

This study provided the first account of cMMN amplitudes in response to a novel dual rule cMMN paradigm that breaks two abstract pattern rules simultaneously. We found significantly reduced cMMN amplitudes in an EPP sample using this paradigm, and the observed effect size of this reduction provides promising evidence that this paradigm may reliably detect auditory processing deficits that can be used as biomarkers for psychosis vulnerability in future predictive models. Moving forward, the cMMN response elicited by the dual rule paradigm should be further examined in EPP and first episode psychosis samples.
